# A case of primary optic pathway demyelination caused by oncocytic oligodendrogliopathy of unknown origin

**DOI:** 10.1186/s40478-022-01462-0

**Published:** 2022-11-08

**Authors:** Simon Hametner, Sara Silvaieh, Majda Thurnher, Assunta Dal-Bianco, Hakan Cetin, Markus Ponleitner, Karin Zebenholzer, Berthold Pemp, Siegfried Trattnig, Karl Rössler, Thomas Berger, Hans Lassmann, Johannes A. Hainfellner, Gabriel Bsteh

**Affiliations:** 1 Division of Neuropathology and Neurochemistry, Department of Neurology, Medical University of Vienna, Vienna, Austria; 2 Department of Neurology, Medical University of Vienna, Vienna, Austria; 3 Biomedical Imaging and Image-Guided Therapy, Division of Neuroradiology and Musculoskeletal Radiology, Medical University of Vienna, Vienna, Austria; 4 Department of Ophthalmology, Medical University of Vienna, Vienna, Austria; 5 High Field MR Center, Department of Biomedical Imaging and Image Guided Therapy, Medical University of Vienna, Vienna, Austria; 6 Department of Neurosurgery, Medical University of Vienna, Vienna, Austria; 7 Center for Brain Research, Medical University of Vienna, Vienna, Austria; 8 Comprehensive Center for Clinical Neurosciences & Mental Health, Medical University of Vienna, Waehringer Guertel 18-20, 1090 Vienna, Austria

**Keywords:** Demyelination, Mitochondria, Oligodendrocytes, Oncocytes

## Abstract

We report the case of a 22-year-old woman presenting with an acute onset of dizziness, gait dysbalance and blurred vision. Magnetic resonance imaging included 3 Tesla and 7 Tesla imaging and revealed a T2-hyperintense, T1-hypointense, non-contrast-enhancing lesion strictly confined to the white matter affecting the right optic radiation. An extensive ophthalmologic examination yielded mild quadrantanopia but no signs of optic neuropathy. The lesion was biopsied. The neuropathological evaluation revealed a demyelinating lesion with marked tissue vacuolization and granular myelin disintegration accompanied by mild T cell infiltration and a notable absence of myelin uptake by macrophages. Oligodendrocytes were strikingly enlarged, displaying oncocytic characteristics and showed cytoplasmic accumulation of mitochondria, which had mildly abnormal morphology on electron microscopy. The diagnosis of multiple sclerosis was excluded. Harding's disease, a variant of Leber's hereditary optic neuropathy, was then suspected. However, neither PCR for relevant mutations nor whole exome sequencing yielded known pathogenetic mutations in the patient's genome. We present a pattern of demyelinating tissue injury of unknown etiology with an oncocytic change of oligodendrocytes and a lack of adequate phagocytic response by macrophages, which to the best of our knowledge, has not been described before.

## Case report

A 22-year-old woman consulted the emergency department of the Vienna General Hospital, Medical University of Vienna, because of gait dysbalance and visual disturbances in terms of painless blurred vision in the left visual field, which suddenly occurred during jogging and was accompanied by a bout of epistaxis. The patient reported several similar episodes of painless blurred vision in the preceding weeks that she attributed to panic attacks. The neurological examination showed a mild physiological anisocoria and gait imbalance (fall propensity to the left side). The past medical history included episodes of depression and panic attacks without the need for regular medication. She reported that she was a non-smoker and not to consume alcohol or illicit drugs. The family history was unremarkable except for an unspecified brain tumor of her father.

**Fig. 1:**
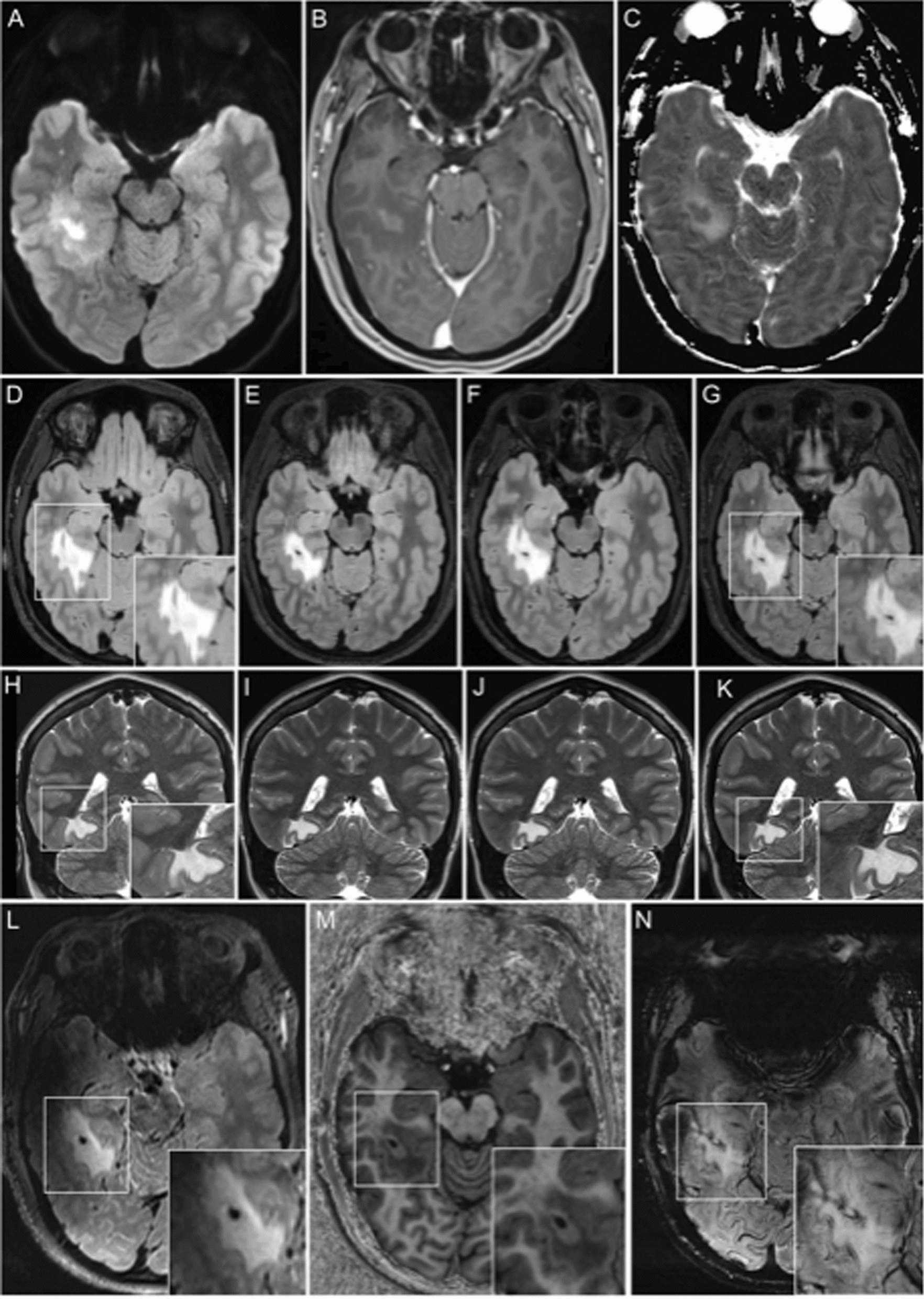
MRI at 3 (**A**–**K**) and 7 Tesla (**L**–**N**). **A**–**C** Initial MRI study. **A** The FLAIR image shows a hyperintense lesion in the right temporal lobe with a strongly hyperintense center and a perilesional zone of intermediate hyperintensity. **B** Post-contrast T1 image revealed slight contrast enhancement in the lesion center and T1-hypointensity in the perilesional zone, most likely corresponding to perilesional edema. **C** Diffusion map with facilitated diffusion in the perilesional area and largely unaltered diffusion metrics in the lesion center. **D–G** Serial axial FLAIR images and **H–K** serial coronal T2-weighted images after biopsy show a largely stable lesion which remains strictly confined to the white matter. Two biopsy sites are visible in the lesion. **L** 7 Tesla FLAIR image reveals a lesion situated in the white matter and partly sparing the subcortical U fibers. **M** T1-weighted MP2RAGE image identifies a homogeneously hypointense lesion area. The biopsy site is surrounded by an area of increased signal, which might be related to the biopsy procedure. **N** SWI shows the biopsy site within a lesion of homogenous high signal intensity. The biopsy site itself is characterized by patchy hypointense signal alterations, likely reflecting extravasation of blood products due to the biopsy

Brain MRI at admission showed a finger-shaped T2- and FLAIR-hyperintense, T1-hypointense lesion in the right temporodorsal region affecting the right optic radiation. The lesion was strictly confined to the white matter and displayed a slight central contrast enhancement without noticeable changes in diffusion metrics (Fig. 1).

Cerebrospinal fluid (CSF) analyses revealed presence of oligoclonal bands with normal IgG/Albumin ratio and a slightly elevated cell count of 6/µl. Ten days later, a follow-up lumbar puncture showed lymphocytic pleocytosis (51/µl) with several macrophages, still a normal IgG/Albumin ratio and positive oligoclonal bands. Further CSF/serum analyses, including onconeural and antineuronal antibodies, antibodies against myelin oligodendrocyte glycoprotein (MOG) and aquaporin-4 (AQP-4), neurotropic viruses, particularly human immunodeficiency virus (HIV) and John Cunningham virus (JCV), yielded negative results.

An 18F-FET (O-(2- [18F] fluoroethyl)-L-tyrosine) PET-CT scan to differentiate between low-grade glioma and a highly suspicious inflammatory lesion showed no elevated amino acid metabolic activity, thus arguing against the presence of a brain tumor. Whole-body 18F-FDG (2-deoxy-2-[18F] fluoro-D-glucose)-PET-(low-dose) CT scan did not show any extra-cerebral abnormalities.

**Fig. 2:**
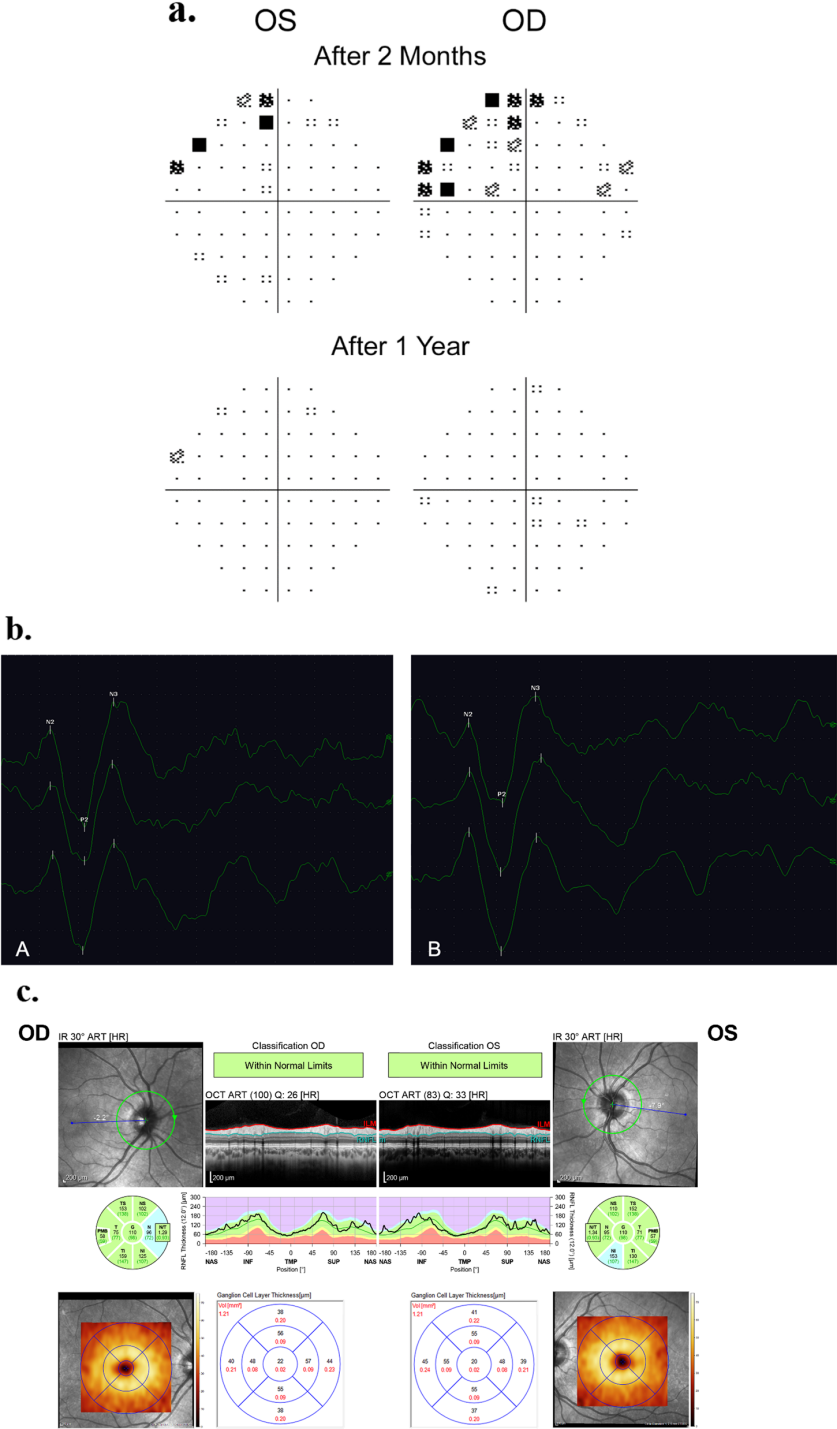
**a** Visual field during follow up. Total deviation probability plots from Humphrey 30–2 tests displaying homonymous visual field defects in the superior left quadrants after 2 months (top) and normal results after 12 months (bottom) (OD = right eye, OS = left eye). **b** Full-field VEP (baseline) 12 × 16 1ch, 5 μV/D, 50 ms/D **(A)** right eye: P2 latency 106 ms, amplitude (P2-N3) 13.6 μV **(B)** left eye: P2 latency 105 ms, amplitude (P2-N3) 12.8 μV. **c** OCT after 12 months Measurement of peripapillary retinal nerve fiber layer (RNFL) thickness (top) and macular ganglion cell layer thickness (bottom). Normal results in both eyes (average RNFL thickness: 110 µm, average ganglion cell layer thickness: 43 µm) with no changes compared to baseline (OD = right eye, OS = left eye)

A detailed ophthalmologic examination including optical coherence tomography (OCT) initially yielded normal findings, in particular no signs of optic neuropathy. After two months, visual field examination showed a mild left superior quadrantanopia (Fig. 2a). Visual evoked potentials (VEP) showed normal results (Fig. 2b). At a follow-up investigation after one year OCT displayed no signs of degeneration (Fig. 2c), and visual field had normalized (Fig. 2a).

**Fig. 3:**
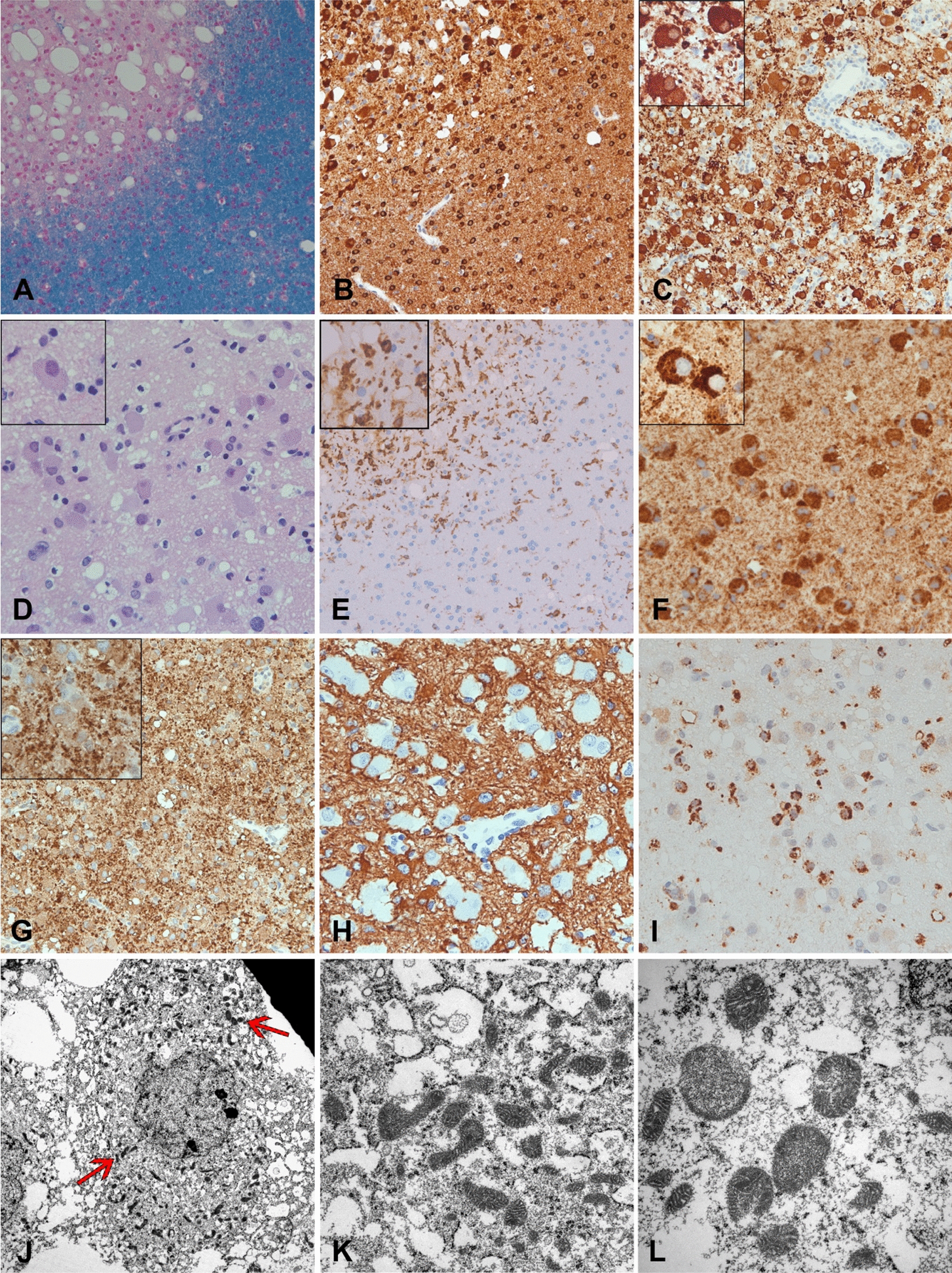
**A** Klüver-Barrera-PAS (KL-PAS) myelin staining shows a focal, sharply demarcated demyelinated white matter lesion with profound vacuolization. **B** Immunohistochemistry (IHC) for the oligodendrocyte marker TPPP/p25 reveals preservation of oligodendrocyte cell bodies in the lesion, which were markedly enlarged when compared to surrounding white matter oligodendrocytes. **C** Same staining as **B** shows profound enlargement of virtually all preserved oligodendrocytes in the lesion. Note the moderate perivascular lymphocyte cuff in the lesion. **D** Hematoxylin & Eosin staining reveals round, enlarged oxyphilic oligodendrocytes with distended eosinophilic, finely granular cytoplasm and round, enlarged and dented nuclei, which are frequently pushed to the cell margin. Mitotic figures or apoptoses are not evident. **E** Immunohistochemistry (IHC) for HLA-DR shows microglia and minor macrophage activation, which was confined to the actual lesion area. **F** VDAC1 IHC targeted against the mitochondrial porin antigen reveals accumulation of mitochondria in the enlarged glial cells. **G** Myelin basic protein IHC shows active granular myelin disintegration. Myelin remnants are found in the extracellular space but not within macrophage cytoplasmata. **H** GFAP staining shows fibrillar gliosis and the absence of immunoreactivity in the enlarged oncocytic glia cells. **I** CD68 IHC displays microglia and small macrophages in a moderate density. Foamy macrophages can hardly be observed in this lesion. **J** Electron micrograph presents a glial cell with round nucleus, large cytoplasm and increased numbers of mitochondria (red arrows). **K** At higher magnification of the glial cytoplasm, the high density of mitochondria is appreciated. **L** Mitochondria frequently displayed unspecific morphological abnormalities in the form of densely packed cristae. Magnifications: **A**, **B**, **C**, **E**, **G**: 200x; **D**, **H**, **I**, Inset in **E**: 400x; All other insets: 600x; **J**: 7000x; **K**: 12000x; **L**: 20000x

As the above-mentioned temporodorsal lesion could not be definitely classified by neuroimaging, a stereotactic brain biopsy was performed. The biopsy contained several informative punches of white matter with unusual and, to the best of our knowledge, unique demyelinating pathological changes, which are presented in Fig. 3. The lesion showed demyelination with profound granular myelin disintegration and relatively well-preserved axonal profiles in the lesion. Inflammatory infiltrates consisted mainly of CD4- and CD8-positive lymphocytes and were moderate in the perivascular cuffs and sparse in the lesion parenchyma. CD20-positive B cells were only rarely found and restricted to perivenous spaces. Moderate microglia activation and moderate macrophage density could be detected. However, macrophages were small, with notably sparse phagocytic activity. Astrocytes displayed fibrillary gliosis. Oligodendrocytes showed unique and unusual morphology, with distended round cell bodies and granular cytoplasm, indicative of an oncocytic change of the oligodendrocytes. Accordingly, the distended cytoplasm showed strong voltage-dependent anion-selective channel 1(VDAC1) positivity, a marker of mitochondria. Increased numbers of mitochondria could be confirmed on the electron microscopy level. Some had slightly abnormal morphology, with an increased numbers of cristae and occasional electron-dense inclusions, which however did not fulfil the morphological criteria of paracrystalline inclusions. In a further step, molecular screening of the mitochondrial DNA for 13 primary and 2 secondary Leber’s hereditary optic neuropathy (LHON) mutations by polymerase chain reaction (PCR) and Sanger sequencing was negative. Further genetic testing using whole-exome sequencing (WES) did not retrieve any known disease-causing mutations in the nuclear genome.

No further symptoms occurred during a follow-up period of 12 months,, while the left superior quadrantanopia improved in visual field testing and was not subjectively perceived anymore after six months. Quarterly MRIs scans did not reveal any relevant changes (including a 7-Tesla brain MRI with no iron accumulation/rims) (Fig. 1).

As the patient remained clinically stable without evidence of disease progression, she neither received immunomodulatory nor any other supportive therapy.

## Discussion

Here, we report a case of primary post-chiasmatic optic pathway demyelination with underlying, pathologically confirmed oligodendrogliopathy. The remarkable morphological changes of oligodendrocytes were accompanied by increased numbers of mitochondria suggestive of an oncocytic change of the oligodendrocytes in the lesion. The oncocytic change, also known as oxyphilic cell transformation, is best known from various neoplastic (e.g. renal oncocytoma) [27] and non-neoplastic reactive or aging conditions, particularly in the parathyroid glands [6]. The oncocytic change has been linked to mitochondrial proliferation as a reaction to mitochondrial NADH dehydrogenase dysfunction or cytochrome c oxidase deficiency [26]. An oncocytic change of glial cells has furthermore been described for neoplastic glial cells in astrocytoma and glioblastoma [24, 38] but not oligodendroglioma. To the best of our knowledge, this is the first report of an oncocytic change of oligodendrocytes in the human brain. Also, we are not aware of an animal model leading to oncocytic changes of oligodendrocytes. Particularly, the cuprizone animal model, which comprises a toxic mitochondrial injury to oligodendrocytes, leads to delayed oligodendrocyte apoptosis but not oncocytic changes [44]. In the current patient’s lesion, the oncocytic changes were strictly confined to oligodendrocytes and associated with a focal lesion of primary demyelination. Besides this unique feature of oligodendrocyte pathology, a further characteristic and unusual aspect of the lesion was the profound accumulation of myelin debris in the extracellular space with only sparse macrophage infiltration and myelin phagocytosis in macrophages, a pattern of demyelination which is atypical for inflammatory demyelinating diseases and further suggests that the destruction of myelin in this condition triggers only minor, probably inadequate activation of macrophages and microglia.

There are various disease entities described as “oligodendrogliopathies”, such as multiple system atrophy (Papp-Lantos inclusions) [41], genetically defined subtypes of amyotrophic lateral sclerosis [28] or single case reports with particular crystalloid oligodendroglial inclusions in a patient with hereditary spastic paraplegia [42]. Globular glial tauopathy is a neurodegenerative condition with 4R tau inclusions in neurons, astrocytes and oligodendrocytes in white and grey matter, white matter myelin loss and upregulation of the mitochondrial carrier MPC1 in white matter glia [8]. However, affected patients are typically older than 40 years and present with symptoms of motor neuron disease or frontotemporal dementia [1], which were not present in our patient. Multiple sclerosis (MS), an immune-mediated chronic-inflammatory demyelinating disease of the central nervous system, is also discussed as a primary oligodendrogliopathy [34] in at least some variants. Mitochondrial damage has been implicated in oligodendroglial injury and subsequent demyelination in a subset of MS patients and rodent models [20, 23, 45]. In our case, by the above-mentioned clinical, neuropathological and neuroimaging findings, we exclude the diagnosis of MS. Pathologically, there was an absence of myelin degradation products in macrophages and astrocytic changes typical of active MS lesions, such as Creutzfeldt-Peters cells. Radiologically, the involvement of only white matter and sparing of U fibers revealed by MRI argues against MS. Moreover, there was no clinical or neuroimaging evidence of CNS inflammation disseminated in time or space over one year of follow-up.

The association between mitochondrial functionality and the integrity of oligodendrocytes and myelin is substantiated by mitochondrial DNA (mtDNA) point mutations causing LHON. This disease is characterized by bilateral optic neuropathy, presenting as acute or subacute, often sequential severe visual loss followed by optic atrophy. While LHON mainly affects men and is typically limited to the anterior optic pathway, there are rare cases where LHON may be accompanied by or manifest as a demyelinating disease very similar to MS, referred to as “Harding’s disease” [9, 12]. Harding’s disease can present itself similar to LHON. Still, it more often affects women (2.1:1) with a higher proportion of patients suffering from more than two visual events or persisting unilateral visual loss, and a longer time interval before the affection of the second eye (average 1.7 years, up to 17 years vs. 0.6 years in LHON) [32]. However, CSF and MRI show changes typical for MS [25]. Post mortem, destructive demyelinating white matter lesions and vacuolating myelin alterations have been documented neuropathologically in a female patient with LHON [18]. In our current patient, a diagnosis of LHON could not be established since there were neither clinical nor paraclinical signs of optic neuropathy (normal VEP, normal OCT, no MRI changes of optic nerve). Furthermore, neither confirmed primary LHON mutations in the mtDNA nor other genetic causes for LHON-like disease in the nuclear DNA could be detected [10, 11, 13, 14, 21, 22, 37, 39]. Still, the follow-up interval of approximately one year might be too short to evaluate the occurrence of clinical signs of Harding’s disease in our patient as the neuropathological findings (see above) would be compatible with mitochondrial dysfunction. Spongy vacuolation of myelin in the CNS white matter, described in our patient, can be found in other diseases involving mitochondrial respiration, such as Kearns-Sayre syndrome or Leigh syndrome [29]. However, our patient did not feature symptoms like progressive external ophthalmoplegia, pigmentary retinopathy, cardiac conduction disorders, deafness or ataxia as described in Kearns-Sayre syndrome [19]. Furthermore, there were no clinical signs pointing at Leigh syndrome, namely developmental delay, ataxia, external ophthalmoplegia, seizures or dystonia [33]. Canavan disease shows spongy vacuolation of subcortical white matter due to a defective gene encoding aspartoacylase, which is particularly enriched in oligodendrocytes. However, onset beyond the age of 5 years (juvenile form of Canavan disease) has not been reported until now to the best of our knowledge [40].

The selective involvement of white matter with profound vacuolization of the tissue could still represent an early stage of a mitochondrial leukodystrophy [29], which may manifest in adulthood [17]. However, the asymmetrical involvement of the brain would not be typical for a leukodystrophy, although the field of genetically defined mitochondrial leukodystrophies continues to expand [35]. Moreover, for some recently described genetically defined leukodystrophies with adult onset, neuropathological correlation was not available or has not been reported [4, 36].

Mutations of genes encoding different mitochondrial proteins, such as OPA1 and POLG1, have been reported in patients with symptoms, neuroimaging findings and oligoclonal bands, all suggestive of MS [7, 43]. OPA1 mutations are the most frequent cause of autosomal dominant optic atrophy (ADOA), characterized by an indolent, slowly progressive, bilateral, symmetric loss of retinal ganglion cells, leading to moderate visual loss. It typically presents with dyschromatopsia, central scotomas and temporal optic atrophy mostly under an age of 20 years [2, 5, 15, 16]. POLG1 mutations may lead to chronic progressive external ophthalmoplegia (CPEO), manifesting with painless bilateral ptosis and ophthalmoplegia [30]. However, our patient did not show any symptoms suggestive of ADOA or CPEO.

In our patient, we found a slight CSF cell count increase from 6/µl to 51/µl after ten days, which could be interpreted as reactive pleocytosis following the repeat lumbar puncture, but may also reflect resorptive response after minor tissue injury. This is is emphasized by the presence of activated macrophages in the CSF. Furthermore, oligoclonal bands were present in CSF. Oligoclonal bands indicate immunoglobulin synthesis in the CNS and are used as biomarkers in inflammatory CNS disorders. They are highly sensitive for subacute and chronic inflammatory diseases but quite unspecific as they occur in a variety of infectious (e.g. neurosyphilis, neuroborreliosis), autoimmune (e.g. multiple sclerosis, autoimmune encephalitis, systemic lupus erythematosus, Sjogren’s syndrome) and other diseases (e.g. brain tumors), and are even found in healthy people [3, 31]. In our case, the presence of oligoclonal bands in CSF could be considered a sign of either a chronic-inflammatory disorder with unnoticed earlier symptoms or an epiphenomenon as part of the oliogendrogliopathy. In particular, the possibility of relapsing chronic inflammatory CNS disease renders our one-year follow-up interval a major limitation of the current report. Therefore, unequivocal interpretation of the presented findings requires further comprehensive follow-up of the patient.

## Conclusion

Here, we report a case of a 22-year old female patient with mild visual field defects caused by demyelination of the post-chiasmatic optic pathway with underlying oncocytic oligodendrogliopathy. Longitudinal clinical and neuroimaging assessment over 12 months currently indicates a monophasic course of the disease. After excluding various inflammatory, infectious and malignant causes, we suggest a metabolic cause, in terms of a mitochondrial disease, as symptoms occurred during physical exercise and neuropathology revealed focal lesions of spongy vacuolation of the white matter with oncocytic enlargement of oligodendrocytes. We propose that this case does not fit into any known neuropathological pattern.

## Data Availability

The datasets used and/or analysed during the current study available from the corresponding author on reasonable request.

## References

[1] Ahmed Z, Doherty KM, Silveira-Moriyama L, Bandopadhyay R, Lashley T, Mamais A, Hondhamuni G, Wray S, Newcombe J, O'Sullivan SS (2011). Globular glial tauopathies (GGT) presenting with motor neuron disease or frontotemporal dementia: an emerging group of 4-repeat tauopathies. Acta Neuropathol.

[2] Alexander C, Votruba M, Pesch UEA, Thiselton DL, Mayer S, Moore A, Rodriguez M, Kellner U, Leo-Kottler B, Auburger G (2000). OPA1, encoding a dynamin-related GTPase, is mutated in autosomal dominant optic atrophy linked to chromosome 3q28. Nat Genet.

[3] Bourahoui A, De Seze J, Guttierez R, Onraed B, Hennache B, Ferriby D, Stojkovic T, Vermersch P (2004). CSF isoelectrofocusing in a large cohort of MS and other neurological diseases. Eur J Neurol.

[4] Chen S, Zou JL, He S, Li W, Zhang JW, Li SJ (2022). Adult-onset autosomal dominant leukodystrophy and neuronal intranuclear inclusion disease: lessons from two new Chinese families. Neurol Sci.

[5] Delettre C, Lenaers G, Griffoin J-M, Gigarel N, Lorenzo C, Belenguer P, Pelloquin L, Grosgeorge J, Turc-Carel C, Perret E (2000). Nuclear gene OPA1, encoding a mitochondrial dynamin-related protein, is mutated in dominant optic atrophy. Nat Genet.

[6] Ding Y, Zou Q, Jin Y, Zhou J, Wang H (2020). Relationship between parathyroid oxyphil cell proportion and clinical characteristics of patients with chronic kidney disease. Int Urol Nephrol.

[7] Echaniz-Laguna A, Chassagne M, de Seze J, Mohr M, Clerc-Renaud P, Tranchant C, Mousson de Camaret B (2010). POLG1 variations presenting as multiple sclerosis. Arch Neurol.

[8] Ferrer I, Andres-Benito P, Zelaya MV, Aguirre MEE, Carmona M, Ausin K, Lachen-Montes M, Fernandez-Irigoyen J, Santamaria E, Del Rio JA (2020). Familial globular glial tauopathy linked to MAPT mutations: molecular neuropathology and seeding capacity of a prototypical mixed neuronal and glial tauopathy. Acta Neuropathol.

[9] Harding AE, Sweeney MG, Miller DH, Mumford CJ, Kellar-Wood H, Menard D, McDonald WI, Compston DA (1992). Occurrence of a multiple sclerosis-like illness in women who have a Leber's hereditary optic neuropathy mitochondrial DNA mutation. Brain.

[10] Howell N, McCullough D, Bodis-Wollner I (1992). Molecular genetic analysis of a sporadic case of Leber hereditary optic neuropathy. Am J Hum Genet.

[11] Huoponen K, Vilkki J, Aula P, Nikoskelainen EK, Savontaus ML (1991). A new mtDNA mutation associated with Leber hereditary optic neuroretinopathy. Am J Hum Genet.

[12] Jansen PH, van der Knaap MS, de Coo IF (1996). Leber's hereditary optic neuropathy with the 11 778 mtDNA mutation and white matter disease resembling multiple sclerosis: clinical, MRI and MRS findings. J Neurol Sci.

[13] Johns DR, Neufeld MJ, Park RD (1992). An ND-6 mitochondrial DNA mutation associated with Leber hereditary optic neuropathy. Biochem Biophys Res Commun.

[14] Jurkute N, Majander A, Bowman R, Votruba M, Abbs S, Acheson J, Lenaers G, Amati-Bonneau P, Moosajee M, Arno G (2019). Clinical utility gene card for: inherited optic neuropathies including next-generation sequencing-based approaches. Eur J Hum Genet.

[15] Kjer P (1959). Infantile optic atrophy with dominant mode of inheritance: a clinical and genetic study of 19 Danish families. Acta Ophthalmol Suppl.

[16] Kline LB, Glaser JS (1979). Dominant optic atrophy. The clinical profile. Arch Ophthalmol.

[17] Kohler W, Curiel J, Vanderver A (2018). Adulthood leukodystrophies. Nat Rev Neurol.

[18] Kovacs GG, Hoftberger R, Majtenyi K, Horvath R, Barsi P, Komoly S, Lassmann H, Budka H, Jakab G (2005). Neuropathology of white matter disease in Leber's hereditary optic neuropathy. Brain.

[19] Lestienne P, Ponsot G (1988). Kearns-Sayre syndrome with muscle mitochondrial DNA deletion. Lancet.

[20] Lucchinetti C, Bruck W, Parisi J, Scheithauer B, Rodriguez M, Lassmann H (2000). Heterogeneity of multiple sclerosis lesions: implications for the pathogenesis of demyelination. Ann Neurol.

[21] Mackey DA, Oostra RJ, Rosenberg T, Nikoskelainen E, Bronte-Stewart J, Poulton J, Harding AE, Govan G, Bolhuis PA, Norby S (1996). Primary pathogenic mtDNA mutations in multigeneration pedigrees with Leber hereditary optic neuropathy. Am J Hum Genet.

[22] Macmillan C, Kirkham T, Fu K, Allison V, Andermann E, Chitayat D, Fortier D, Gans M, Hare H, Quercia N (1998). Pedigree analysis of French Canadian families with T14484C Leber's hereditary optic neuropathy. Neurology.

[23] Mahad D, Ziabreva I, Lassmann H, Turnbull D (2008). Mitochondrial defects in acute multiple sclerosis lesions. Brain.

[24] Marucci G, Maresca A, Caporali L, Farnedi A, Betts CM, Morandi L, de Biase D, Cerasoli S, Foschini MP, Bonora E (2013). Oncocytic glioblastoma: a glioblastoma showing oncocytic changes and increased mitochondrial DNA copy number. Hum Pathol.

[25] Matthews L, Enzinger C, Fazekas F, Rovira A, Ciccarelli O, Dotti MT, Filippi M, Frederiksen JL, Giorgio A, Kuker W (2015). MRI in Leber's hereditary optic neuropathy: the relationship to multiple sclerosis. J Neurol Neurosurg Psych.

[26] Muller-Hocker J, Schafer S, Krebs S, Blum H, Zsurka G, Kunz WS, Prokisch H, Seibel P, Jung A (2014). Oxyphil cell metaplasia in the parathyroids is characterized by somatic mitochondrial DNA mutations in NADH dehydrogenase genes and cytochrome c oxidase activity-impairing genes. Am J Pathol.

[27] Ng KL, Morais C, Bernard A, Saunders N, Samaratunga H, Gobe G, Wood S (2016). A systematic review and meta-analysis of immunohistochemical biomarkers that differentiate chromophobe renal cell carcinoma from renal oncocytoma. J Clin Pathol.

[28] Nolan M, Barbagallo P, Turner MR, Keogh MJ, Chinnery PF, Talbot K, Ansorge O (2021). Isolated homozygous R217X OPTN mutation causes knock-out of functional C-terminal optineurin domains and associated oligodendrogliopathy-dominant ALS-TDP. J Neurol Neurosurg Psychiatry.

[29] Oldfors A, Tulinius M (2003). Mitochondrial encephalomyopathies. J Neuropathol Exp Neurol.

[30] Petty RK, Harding AE, Morgan-Hughes JA (1986). The clinical features of mitochondrial myopathy. Brain.

[31] Petzold A (2013). Intrathecal oligoclonal IgG synthesis in multiple sclerosis. J Neuroimmunol.

[32] Pfeffer G, Burke A, Yu-Wai-Man P, Compston DA, Chinnery PF (2013). Clinical features of MS associated with Leber hereditary optic neuropathy mtDNA mutations. Neurology.

[33] Rahman S, Blok RB, Dahl HH, Danks DM, Kirby DM, Chow CW, Christodoulou J, Thorburn DR (1996). Leigh syndrome: clinical features and biochemical and DNA abnormalities. Ann Neurol.

[34] Rone MB, Cui QL, Fang J, Wang LC, Zhang J, Khan D, Bedard M, Almazan G, Ludwin SK, Jones R (2016). Oligodendrogliopathy in multiple sclerosis: low glycolytic metabolic rate promotes oligodendrocyte survival. J Neurosci.

[35] Roosendaal SD, van de Brug T, Alves C, Blaser S, Vanderver A, Wolf NI, van der Knaap MS (2021). Imaging patterns characterizing mitochondrial leukodystrophies. AJNR Am J Neuroradiol.

[36] Sarkar P, Mukherjee A, Sarkar S, Agrawal R, Dubey S, Pandit A (2022). Adult-onset dystonia with late-onset epilepsy in TUBB4A-related hypomyelinating leukodystrophy-a new intermediate phenotype. Ann Indian Acad Neurol.

[37] Stenton SL, Sheremet NL, Catarino CB, Andreeva NA, Assouline Z, Barboni P, Barel O, Berutti R, Bychkov I, Caporali L (2021). Impaired complex I repair causes recessive Leber's hereditary optic neuropathy. J Clin Invest.

[38] Tsunoda S, Sakaki T, Kubota T, Goda K, Nakamura M, Hashimoto H, Hoshida T, Morimoto T (1992). Anaplastic astrocytoma of an oncocytic type occurring in the cerebellar vermis in Pierre Robin syndrome–case report. Neurol Med Chir (Tokyo).

[39] Wallace DC, Singh G, Lott MT, Hodge JA, Schurr TG, Lezza AM, Elsas LJ, Nikoskelainen EK (1988). Mitochondrial DNA mutation associated with Leber's hereditary optic neuropathy. Science.

[40] Wei H, Moffett JR, Amanat M, Fatemi A, Tsukamoto T, Namboodiri AM, Slusher BS (2022). The pathogenesis of, and pharmacological treatment for Canavan disease. Drug Discov Today.

[41] Wenning GK, Stefanova N, Jellinger KA, Poewe W, Schlossmacher MG (2008). Multiple system atrophy: a primary oligodendrogliopathy. Ann Neurol.

[42] Woehrer A, Laszlo L, Finsterer J, Stollberger C, Furtner J, Rinner W, Molnar K, Budka H, Kovacs GG (2012). Novel crystalloid oligodendrogliopathy in hereditary spastic paraplegia. Acta Neuropathol.

[43] Yu-Wai-Man P, Spyropoulos A, Duncan HJ, Guadagno JV, Chinnery PF (2016). A multiple sclerosis-like disorder in patients with OPA1 mutations. Ann Clin Transl Neurol.

[44] Zendedel A, Beyer C, Kipp M (2013). Cuprizone-induced demyelination as a tool to study remyelination and axonal protection. J Mol Neurosci.

[45] Ziabreva I, Campbell G, Rist J, Zambonin J, Rorbach J, Wydro MM, Lassmann H, Franklin RJ, Mahad D (2010). Injury and differentiation following inhibition of mitochondrial respiratory chain complex IV in rat oligodendrocytes. Glia.

